# GLUT5 (*SLC2A5*) enables fructose-mediated proliferation independent of ketohexokinase

**DOI:** 10.1186/s40170-021-00246-9

**Published:** 2021-03-24

**Authors:** Roger J. Liang, Samuel Taylor, Navid Nahiyaan, Junho Song, Charles J. Murphy, Ezequiel Dantas, Shuyuan Cheng, Ting-Wei Hsu, Shakti Ramsamooj, Rahul Grover, Seo-Kyoung Hwang, Bryan Ngo, Lewis C. Cantley, Kyu Y. Rhee, Marcus D. Goncalves

**Affiliations:** 1 Division of Endocrinology, Weill Department of Medicine, Weill Cornell Medicine, New York, NY 10065 USA; 2 Meyer Cancer Center, Department of Medicine, Weill Cornell Medicine, New York, NY 10065 USA; 3 Weill Cornell Graduate School of Medical Sciences, Weill Cornell Medicine, New York, NY 10065 USA; 4 Weill Cornell/Rockefeller/Sloan Kettering Tri-I MD-PhD program, New York, NY 10065 USA; 5 Division of Infectious Diseases, Weill Department of Medicine, Weill Cornell Medicine, New York, NY 10065 USA; 6 Center for Molecular Oncology, Memorial Sloan Kettering Cancer Center, New York, NY 10065 USA; 7 Department of Pathology, Memorial Sloan Kettering Cancer Center, New York, NY 10065 USA; 8 Weill Cornell Medical College, Weill Cornell Medicine, New York, NY 10065 USA

**Keywords:** Fructose, Ketohexokinase, Hexokinase, GLUT5 (SLC2A5), Metabolism

## Abstract

**Background:**

Fructose is an abundant source of carbon and energy for cells to use for metabolism, but only certain cell types use fructose to proliferate. Tumor cells that acquire the ability to metabolize fructose have a fitness advantage over their neighboring cells, but the proteins that mediate fructose metabolism in this context are unknown. Here, we investigated the determinants of fructose-mediated cell proliferation.

**Methods:**

Live cell imaging and crystal violet assays were used to characterize the ability of several cell lines (RKO, H508, HepG2, Huh7, HEK293T (293T), A172, U118-MG, U87, MCF-7, MDA-MB-468, PC3, DLD1 HCT116, and 22RV1) to proliferate in fructose (i.e., the fructolytic ability). Fructose metabolism gene expression was determined by RT-qPCR and western blot for each cell line. A positive selection approach was used to “train” non-fructolytic PC3 cells to utilize fructose for proliferation. RNA-seq was performed on parental and trained PC3 cells to find key transcripts associated with fructolytic ability. A CRISPR-cas9 plasmid containing *KHK*-specific sgRNA was transfected in 293T cells to generate *KHK*^*-/-*^ cells. Lentiviral transduction was used to overexpress empty vector, KHK, or GLUT5 in cells. Metabolic profiling was done with seahorse metabolic flux analysis as well as LC/MS metabolomics. Cell Titer Glo was used to determine cell sensitivity to 2-deoxyglucose in media containing either fructose or glucose.

**Results:**

We found that neither the tissue of origin nor expression level of any single gene related to fructose catabolism determine the fructolytic ability. However, cells cultured chronically in fructose can develop fructolytic ability. *SLC2A5*, encoding the fructose transporter, GLUT5, was specifically upregulated in these cells. Overexpression of GLUT5 in non-fructolytic cells enabled growth in fructose-containing media across cells of different origins. GLUT5 permitted fructose to flux through glycolysis using hexokinase (HK) and not ketohexokinase (KHK).

**Conclusions:**

We show that GLUT5 is a robust and generalizable driver of fructose-dependent cell proliferation. This indicates that fructose uptake is the limiting factor for fructose-mediated cell proliferation. We further demonstrate that cellular proliferation with fructose is independent of KHK.

**Supplementary Information:**

The online version contains supplementary material available at 10.1186/s40170-021-00246-9.

## Background

Fructose is an important contributor to cell metabolism, growth, and disease. It is the second most abundant sugar in the blood and is commonly consumed as part of the Western diet. Most caloric sweeteners including sucrose, honey, and high-fructose corn syrup contain at least 40% fructose, and the yearly consumption of these caloric sweeteners in the USA is over 120 lbs (~60 kg) per capita [1]. The excessive availability of fructose-containing sugars has negatively altered human physiology and predisposed us to cardiometabolic disease, insulin resistance, and obesity [2, 3].

Fructose metabolism is a tissue-specific. Canonical fructose metabolizing organs include the kidney and those found in the gastrointestinal tract such as the liver, pancreas, and intestine. In these organs, fructose enters through the fructose transporter, GLUT5, before being phosphorylated by KHK and cleaved by Aldolase B (ALDOB) into glyceraldehyde and dihydroxyacetone phosphate. Both of the products can be metabolized into glyceraldehyde-3-phosphate, a downstream glycolytic intermediate. Digestive organs are directly exposed to dietary fructose on a daily basis, and they express high levels of fructose metabolism genes [4, 5]. Metabolic tracing experiments have proved that dietary fructose is metabolized to fructose-1-phosphate (F1P) in these organs [6]. However, other organs—such as the heart, muscle, and certain parts of the brain—have also been reported to metabolize fructose [5, 7–11].

Tumors can also metabolize fructose. This has been shown for a variety of tumor types arising from the breast, brain, prostate, ovary, pancreas, intestine, lung, liver, kidney, and blood ( [5, 12]; breast [13, 14], brain [15, 16], prostate [17], ovary (Jin et al., [18]), pancreas [19, 20], intestine [21], lung [22–24], liver [25], kidney [26], and blood [27, 28]). In many of these cases, fructose has been shown to enter the cell through a membrane transporter, GLUT5, and then undergo metabolism into downstream glycolytic intermediates. In tumors, it has been presumed, but not clearly shown, that fructose is metabolized by ketohexokinase. It also remains unclear what basic machinery is required by tumor cells to permit fructose metabolism.

In this study, we set out to determine the cell-intrinsic factors that enable tumor cell proliferation in fructose. We profiled 13 cancer cell lines from 5 different origins and demonstrate that neither the tissue of origin nor expression level of any individual gene related to fructose metabolism determine fructolytic ability. We “trained” non-fructolytic cells in a high-fructose, low-glucose media in order to obtain cells that metabolize fructose. The trained cells showed strong upregulation in the expression of *SLC2A5*, the gene encoding GLUT5. Overexpression of GLUT5 allowed six non-fructolytic cell lines of different origins to proliferate in fructose media. This proliferation did not require KHK. Instead, fructose was preferentially metabolized by hexokinase. Taken together, these findings demonstrate that cells proliferate using fructose by upregulating GLUT5 independent of KHK.

## Methods

### Experimental model and subject details

#### Cell culture

RKO, H508, HepG2, Huh7, HEK293T (293T), A172, U118-MG, U87, MCF-7, MDA-MB-468, and PC3 cells were obtained from ATCC. DLD1 and HCT116 cells were a generous gift from Lukas Dow. 22RV1 and was a generous gift from Dawid Nowak. 22Rv1, PC3, and H508 cells were cultured in full RPMI (Corning, Corning, NY) supplemented with 10% fetal bovine serum (FBS) (Gemini, Sacramento, CA) and 1% penicillin/streptomycin (Life Technologies, Carlsbad, CA). All of the other cells were cultured in DMEM (Corning) supplemented with 10% FBS and 1% penicillin/streptomycin (Life Technologies). HepG2 cells were grown on collagen coated plates (2 ug/cm^2^). Cell lines were STR fingerprinted and/or bought from ATCC directly. Cells were tested for mycoplasma (Lonza, Basel, Switzerland).

Sugarless RPMI (Life Technologies) and DMEM (Life Technologies) were used in many experiments. Glucose (Millipore-Sigma, Burlington, MA) and fructose (St. Louis, MO) powders were diluted to 1 M stock in water before filtration. This stock solution was diluted into sugarless media.

To generate the semi-trained PC3 line, the parental cells were cultured in RPMI (Life Technologies) containing 1 mM glucose, 10 mM fructose, and 5% dFBS (Life Technologies). Cells were passaged approximately once per week. After >20 passages, semi-trained cells were cultured in a 10-mM fructose in order to generate trained PC3 cells.

### Method details

#### RNA extraction, RT-qPCR, and RNA-seq

Total RNA was isolated directly from plates using the RNeasy Mini Kit (Qiagen, Hilden, Germany). For qPCR, 1.25 μg RNA was reversed-transcribed using SuperScript VILO Master Mix (Thermo Fisher, Waltham, MA). Resulting cDNA was diluted 1:10 and qPCR was performed with Fast SYBR Green Mastermix (Life Technologies). The relative expression of each gene was calculated by comparative ΔCt method after normalizing to endogenous controls (Raw dCt in Table S2, Primers in Table S3). A heatmap of the results was produced using the Qlucore Omics Explorer (Qlucore, Lund, Sweden).

RNA samples from PC3 and semi-trained PC3 were submitted to the Weill Cornell Medicine Genomics Core for paired-end RNA-seq on a NovaSeq 6000. Raw sequenced reads were aligned to the mouse reference GRCm38 using STAR (v2.4.1d, 2-pass mode) aligner. Aligned reads were quantified using Cufflinks (v2.2.1) to obtain fragments per kilobase per million (FPKM). Statistical analyses on the normalized expression values (FPKM) were performed using the Qlucore Omics Explorer (Qlucore, Lund, Sweden). Gene expression levels were log_2_ transformed before performing PCA and differential gene expression analysis.

#### Genomic DNA (gDNA) extraction and qPCR

A 500-uL genomic lysis buffer (20 mM Tris-HCl pH 7.5, 20 mM EDTA, 1% SDS, 400 ug/mL proteinase K) was used to lyse 500,000 cells. Proteinase K was heat inactivated at 95°C for 15 min and allowed to cool to room temperature. Protein was precipitated with 5 M NaCl, and sample was centrifuged at 13,000 × rpm at room temperature for 10 min. Supernatant was poured out, and pellet was washed with 1 mL 70% ethanol. Samples were centrifuged for at 13,000 × rpm for 5 min and supernatant was drained. Pellets were resuspended in 10 mM Tris-HCl pH 8.0. To analyze *SLC2A5* copy number, qPCR was performed on 40 ng of gDNA using Fast SYBR Green Mastermix. Primers were designed to be within the same exon for *SLC2A5* and *B2M* and can be found in Table S3.

#### Cell line mutation and clinical data analysis

Cell line genomic data were downloaded from the Cancer Cell Line Encyclopedia (CCLE, Broad Institute) [29] or the COSMIC (Wellcome Sanger Institute) [30] databases and cross-referenced with known oncogenic mutations from COSMIC tier 1 genes (Table S1). The full list of oncogenic mutations for each cell line can be found in Supplementary File 1. Mutation and clinical data for each cell line were cross-referenced with Cellosaurus (Table S1) [31].

#### Cell confluence, viability, and the fructolytic index

Cells were plated at low confluency in a 6- or 12-well dish. After settling, cells received a PBS wash and were given 5% dFBS, 1% penicillin-streptomycin media containing no sugar, 10 mM glucose, or 10 mM fructose. Plates were loaded into IncuCyte ZOOM Live Cell Analysis System (Essen Bioscience, Ann Arbor, MI) for imaging. Sixteen frames per well were analyzed at each timepoint to determine confluency. Change in confluency per hour was measured by linear regression on Prism (Graphpad, San Diego, CA).

Independent cell proliferation experiments were used to produce the fructolytic index (*n* = 3). It was calculated by dividing the relative growth in fructose (growth rate in fructose – growth rate in the no-sugar control) by the relative growth in glucose (growth rate in glucose – growth rate in the no-sugar control). After 3–4 days in the Incucyte system, cells were fixed with ice cold 80% methanol before. Crystal violet reagent (Sigma-Aldrich) was added to each well, and the plates were placed on a rocker for 30 min. Cells were then rinsed with water and imaged with a scanner.

For the competition assay, phase contrast and fluorescent images from the Incucyte system were exported as TIFF files. A custom ImageJ (Bethesda, MD) program (https://github.com/sam-taylor/CompCount) was used to acquire cell count and size. A bandpass filter, automatic threshold, and watershed algorithm were employed to distinguish cells from background. Data from the individual images were compiled into groups using MATLAB (Natick, MA) statistical software.

To measure sensitivity to drugs, cells were plated at low confluency with several replicates in a 96-well white bottom plate. The next day, powdered 2-DG (Sigma-Aldrich) was reconstituted in 10 mM glucose or 10 mM fructose media to make 100 mM 2-DG stock, which was then serially diluted. Cells were washed with PBS and were given 5% dFBS, 1% penicillin-streptomycin media containing either 100 μL of 10 mM glucose or 10 mM fructose media containing serially diluted 2-DG. Cell viability was measured after 72 h using Cell Titer Glo reagent according to manufacturer’s instructions (Promega, Fitchburg, WI). Plates were covered and rocking for 15 min before luminescence was measured.

#### Western blots

Whole cell lysates were extracted with RIPA buffer (CST, Danvers, MA) containing protease and phosphatase inhibitor (Life Technologies) and quantified with BCA reagent (Thermo Fisher). Murine muscle, liver, and *Khk*^*-/-*^ liver was obtained from our previous study [21]. Equal amounts of protein were diluted in 4× LDS buffer (Life Technologies) before being run in 4–12% bis-tris gels (Invitrogen, Carlsbad, CA). Gels were transferred to PVDF membranes (Perkin-Elmer, Waltham, MA) and blocked for 1 h in 5% BSA in Tris-buffered saline containing 1% Tween 20 (TBST). The membranes were probed while rocking at 4°C with the following antibodies and concentrations: GLUT1 (Millipore 07-1401) 1:1000, GLUT2 (Abcam, Cambridge, UK, ab192599) 1:1000, GLUT5 (Invitrogen, PA5-80023) 1:1000, KHK (Abcam) 1:1000, HK1 (CST 2024) 1:1000, HK2 (CST 2867) 1:1000, ALDOA (CST8060) 1:1000, ALDOB (Abcam ab152828) 1:1000, ALDOC (Proteintech, Rosemont, IL, 14884-1-AP) 1:1000, LDHA (CST) 1:1000, LDHB (Abcam) 1:1000, GAPDH (Proteintech 10494-1-AP) 1:5000, Pan-Actin (CST 4968) 1:1000, and V5-HRP (Life Technologies R96125) 1:5000. After incubation, cells were washed with TBST before appropriate HRP-conjugated secondary antibody was added for 1 h. After 3 more TBST washes, the membranes were exposed to Supersignal West Dura (Life Technologies) and imaged using a ChemiDoc MP Imaging System (BioRad, Hercules, CA).

#### Plasmids and cloning

The following plasmids were generously provided by researchers via Addgene: pSpCas9(BB)-2A-Puro (PX459) V2.0 (#62988) from Dr. Feng Zhang (Broad Institute) (Ran et al., 2013) m pDONR221-*SLC2A5* (#132090) from the RESOLUTE Consortium and Giuliu Superti-Furga (Research Center for Molecular Medicine of the Austrian Academy of Sciences), and pLenti-U6-tdTomato-P2A-BlasR (Lrt2B) (#110854) from Dr. Lukas Dow (Weill Cornell Medicine) [32].

We selected sgRNA (Figure S3) for human KHK at the beginning of exon 5 using CRISPRdirect [33]. Oligonucleotide pairs were annealed and cloned into PX459 using BbsI-HF (New England Biolabs, Ipswich, MA) followed by a ligation reaction (New England Biolabs). PDONR221-GLUT5 was cloned according to Gateway Technology (Invitrogen) into pLenti7.3_V5_DEST (Invitrogen) using LR Clonase (Invitrogen) in order to generate pLenti7.3_V5_SLC2A5. These plasmids were generated in Stbl3 bacteria (Life Technologies) and were purified using Qiagen miniprep or maxiprep kits (Qiagen).

### Generating knockouts

We plated 200,000 cells/well in a 6-well dish. The following day, cells were transfected with 3 μL Lipofectamine 2000 (Life Technologies) and 3 ug plasmid containing sgRNA in Optimem (Life Technologies). The following day, media was changed. The day after media change, cells were selected with 2 ug/mL puromycin for 48 h. Fifty or 100 cells were then passaged into 10 cm dishes and were allowed to proliferate into visible colonies over 2 weeks. Single colonies with normal morphology were selected using cloning cylinders (Thermo Fisher). Knockouts were verified by western blot and sanger sequencing.

#### Transduction

2,000,000 293T cells were plated in a 10-cm dish. The next day, cells were transfected with 30 μL Lipofectamine 2000, 9 ug psPAX2, 1 ug VSV-G, and 9 ug of either Lrt2b, pLenti7.3-V5 EV, and pLenti7.3-V5-*SLC2A5.* Media were changed the following day. Viral particles were harvested 48 and 72 h after initial media change. The 2 harvests were combined and aliquoted for storage in −80°C.

To generate PC3-red, parental cells were given 50% Lrt2b virus and 50% media as well as 10 ug/mL polybrene. The next day, cells were given 50% virus and 50% media as well as 10 ug/mL polybrene. Media was changed after 24 h. The day after media change, cells were grown in media containing 10 ug/mL blasticidin (Invivogen, San Diego, CA). Overexpression was verified by microscopy.

To overexpress GLUT5, non-fructolytic cell lines from several origins were plated at low confluence in 6-well dishes. The next day, cells were given 50% EV or *SLC2A5* virus and 50% media as well as 10 ug/mL polybrene. Media was changed after 24 h. Overexpression was verified by western blot.

#### Seahorse assay

ECAR was measured with the Seahorse XFe96 Analyzer (Agilent, Santa Clara, CA), following manufacturer’s Glycolytic Stress Test protocol. Briefly, 5,000 cells were plated in each well of a 96-well Seahorse assay plate. That same day, the assay cartridge was hydrated and kept in a non-CO_2_ incubator at 37°C. After 12–24 h, cells were washed with PBS before they were given reconstituted sugarless DMEM powder (Sigma-Aldrich) supplemented with 2 mM glutamine and 5 mM HEPES buffer. Cells were then incubated for 45 min at 37°C in a non-CO_2_ incubator. Compounds (final concentrations: glucose 10 mM or fructose 10 mM, oligomycin 1 uM, and 2-DG 50 mM) were prepared, loaded into the flux pack, and put into the Seahorse XFe96 Analyzer. The plate containing cells were subsequently loaded into the machine. ECAR was analyzed using Seahorse Wave software.

#### Metabolite extraction, targeted analysis, and untargeted analysis

Metabolomics were carried out on cells to measure polar metabolites. 500,000 cells were plated in triplicate in 6-well dishes for each condition. The next day, cells were washed briefly with 37°C PBS before given media containing no glucose and 10 mM [U-^13^C]-fructose (Cambridge Isotope Laboratories, Tewksbury, MA). After a 30-min incubation, cells were washed briefly with warm PBS and immediately harvested into 2 mL Eppendorf tubes using with ice cold 80% methanol (Yuan et al., 2012) and 0.02 M formic acid. Cells were vortexed and stored in −80 °C overnight. Samples were spun down at 13,000 × RPM for 10 min at 4°C. Supernatant was transferred to a new Eppendorf tube and was evaporated for LC/MS.

Quantitative metabolomics were performed on samples as previously described [21]. Briefly, 5 μL of each filtered extract was injected through an Agilent ZORBAX Extend C18, 2.1 × 150 mm, 1.8 (Agilent) downstream of an Agilent ZORBAX SB-C8, 2.1 mm× 30 mm, 3.5 um guard column (Agilent) heated to 40°C in the Agilent 1290 Infinity LC system. Solvent A (97% water/3% methanol containing 5 mM tetrabutylammonium hydroxide (TBA) and 5.5 mM acetic acid) and Solvent B (methanol containing 5 mM TBA and 5.5 mM acetic acid) were infused at a 0.250 mL/min flow rate. The reverse phase gradient was as follows: 0–3.5 min, 0% B; 4–7.5 min, 30% B; 8–15 min, 35% B; 20–24 min, 99% B; followed by a 7-min run at 0% B. Acquisition was performed on the Agilent 6230 TOF mass spectrometer (Agilent) using an Agilent Jet Stream electrospray ionization source (Agilent) operated at 4000 V Cap and 2000 V nozzle voltage in high-resolution, negative mode. During acquisition, the sample nebulizer was set to 45 psig with sheath gas flow of 12L/min at 400°C. Drying gas was kept at 325°C at 8 L/min. The fragmentor was set to 125 V, with the skimmer set to 50 V and Octopole Vpp at 400 V. Samples were acquired in centroid mode for 1.5 spectra/s for m/z’s from 50–1100.

Data was analyzed by batch processing with Agilent MassHunter Profinder software (Agilent) for both targeted and untargeted analysis. For targeted analysis, we identified metabolites by both retention time and with authentic standards. We identified untargeted compounds using Profinder Batch Targeted Feature Extraction. Then, we processed hits through Agilent Mass Profiler Professional software for quality control.

#### Quantification and statistical analysis

Sample size was estimated based on prior data [21]. Data is presented as ± standard error of the mean (SEM), calculated by Graphpad Prism 8. For total metabolites and GLUT5 rescue growth rates, unpaired two-tail *t* tests were done between control and experimental conditions. For RT-qPCR data and 13C metabolomics, two-way ANOVA was done with post-test comparisons made by Fisher’s LSD test. Statistical significance is indicated in figures using the following denotation: *P < 0.05, **P< 0.01, ***P < 0.001, and ****P < 0.0001. Sample number was noted in figure legends.

#### Software availability

An application to perform cell quantification from images was created by S.T and is available on https://github.com/sam-taylor/CompCount.

## Results

### The fructolytic index quantifies proliferation using fructose relative to glucose

**Fig. 1:**
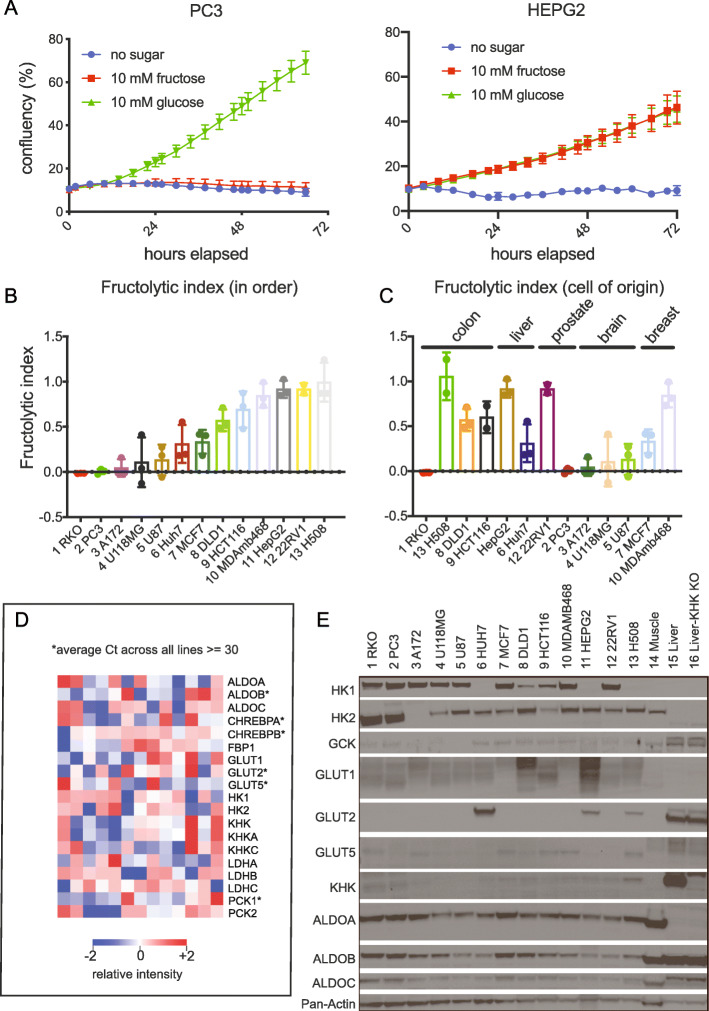
Cellular gene expression and tissue of origin do not determine cellular proliferation in fructose. **a** PC3 and HepG2 were seeded into 12-well plates (20,000 cells/well) and cultured in the absence or presence of 10 mM fructose, or 10 mM glucose media for approximately 3 days. Cell density (% confluency) was monitored over time using live cell imaging (*n =* 2 per media condition). **b** Fructolytic index (fructose-mediated growth/glucose-mediated growth) of the indicated cell lines arranged in order of least to most fructolytic (*n* = 3). **c** Fructolytic index of cell lines in **b** grouped by tissue of origin. **d** Normalized expression of the indicated genes for each cell line shown as a heatmap. Cell lines ordered by fructolytic index (*n* = 2 per gene per cell line). *Denotes C_t_ > 30. **e** Immunoblot of the indicated proteins using lysates from the indicated cell lines, ordered from least to most fructolytic. The murine muscle, liver, and *Khk* knockout liver were used as controls

We measured the ability of 13 tumor cell lines to proliferate in 10 mM fructose and in 10 mM glucose using live cell imaging. Cells were sampled from a variety of organs including the brain, breast, prostate, liver, and colon/rectum. We noticed a striking difference in the ability of cells to proliferate in fructose as determined by live cell imaging (Figure S1A). For example, metastatic prostate PC3 cells do not grow in fructose media, but hepatocellular carcinoma HepG2 cells do (Fig. 1a). We verified these results with a crystal violet assay after 3–4 days of growth (Figure S1B).

To quantify and compare the fructolytic ability among the cells, we created the fructolytic index. This index is calculated by dividing the relative growth in fructose (growth rate in fructose minus growth rate in the no-sugar control) by the relative growth in glucose (growth rate in glucose minus growth rate in the no-sugar control) (Figure S2A). In other words, it is a ratio of how well cells utilize fructose compared to glucose as a growth substrate (Fig. 1b). Of note, we used 5% dialyzed FBS (dFBS) to minimize the contamination of FBS-related sugars to the media. The concentration of dFBS in the culture media was held constant at 5% in all cell lines except for 22RV1, which required 1% in our growth assays (Figure S2B-C).

### Neither the tissue of origin nor expression level of any individual gene related to fructose metabolism determines fructolytic growth

There was heterogeneity in the fructolytic index amongst cells derived from the same tissue (Fig. 1c). We reviewed the genomic mutations and clinical parameters associated with each cell line and found no obvious trend that predicts fructose growth (Table S1). We also profiled the cell lines for their expression of select fructolytic and glycolytic genes and found no clear correlation of any individual transcript or protein with the fructolytic index (Fig. 1d–e, Table S2). Unbiased hierarchical clustering of the samples according to gene expression similarly failed to group the cells by fructolytic index (Figure S2D). Taken together, commonly used methods and existing bioinformatic annotations failed to predict the fructolytic index of cell lines.

### Cells can be trained to proliferate with fructose

**Fig. 2:**
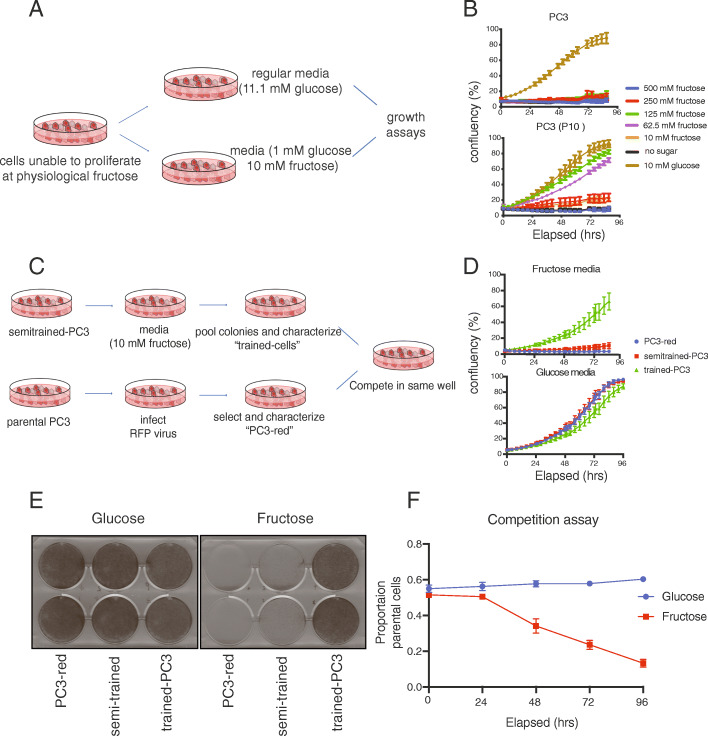
Cells can be trained to metabolize fructose for proliferation. **a** Schematic for the positive selection strategy to generate fructolytic cell lines. **b** PC3 and PC3 passage 10 (P10) cells were seeded into 96-well plate (1500 cells/well) and cultured in media containing various amounts of sugar. Cell density (% confluency) was monitored over time using live cell imaging (*n* = 2 per condition). **c** Schematic for the competition growth assay between PC3-red (parental PC3 cells transduced with RFP reporter) and fructose-trained cell lines. **d** 40,000 of PC3, semi-trained PC3 passage 20 (P20), and trained-PC3 cells in 10 mM fructose or 11 mM glucose over time (*n* = 2 per condition). **e** Cells from **d** were grown in 10 mM fructose or 11 mM glucose for 96 h. They were then fixed and stained with crystal violet solution (*n* = 2 per condition). **f** 20,000 PC3-red and 20,000 trained PC3 cells were seeded in the same well and cultured for 96 h in 10 mM fructose or 10 mM glucose-containing media. Live fluorescent imaging was performed and the proportion of PC3-red cells to total PC3 cells is shown over time (*n* = 2 per condition). Supplemental Video 1 and Supplemental Video 2 are of competition assays monitored with live cell imaging

To determine how cells utilize fructose, we attempted to “train” a non-fructolytic cell line to proliferate using this sugar. We employed a positive selection approach that was inspired by in vitro drug resistance studies, whereby researchers add selective pressure to bacteria or tumor cells in order to find and characterize drug-resistant clones [34, 35] (Fig. 2a). PC3, a cell line with a low fructolytic index, was grown in media containing high fructose and limiting amounts of glucose for several passages. The original PC3 line was cultured with non-fructose containing media in parallel as a control.

By passage 10 (P10), the line grown in fructose gained the ability to proliferate in fructose, albeit only at high concentrations (>62.5 mM) (Fig. 2b). By passage 20 (P20), the cells could proliferate at lower concentrations (>10 mM) of fructose, and we called these cells “semi-trained” (Figure S3A-B). We next removed glucose completely from culture media of the “semi-trained” cell lines in hopes of selecting for cells that best proliferated in fructose (Fig. 2c). Recovered cells initially proliferated slowly, but after 1–2 passages, “trained” cells proliferated equally well in glucose and fructose (Fig. 2d-e, Supplemental video 1).


**Additional file 2: Supplemental Video 1.** Trained PC3 cells outcompete parental PC3 cells in fructose media. 20,000 PC3-red and 20,000 trained PC3 cells were seeded in a 6-well dish containing 10 mM fructose. Cells were monitored with live cell imaging for 4 days.

To control for plating and media conditions, we co-plated the parental PC3 line with the trained cells in a competition assay [36] (Fig. 2c). Parental cells were labeled with an RFP reporter and plated at a 1:1 ratio with trained cells. In glucose-media, the final number of parental and trained cells were equal, but in fructose-media, the parental cells only constituted 10–15% of total cells (Fig. 2f, Supplemental Video 1-2). We next asked if the acquired ability to proliferate with fructose was lost when cells were grown in glucose for long periods of time. Even after 5 passages in media devoid of fructose, the cells completely retained their fructolytic ability (Figure S3C-E).


**Additional file 3: Supplemental Video 2.** Trained PC3 cells grow at the same rate as parental PC3 cells in glucose media. 20,000 PC3-red and 20,000 trained PC3 cells were seeded in a 6-well dish containing 10 mM glucose. Cells were monitored with live cell imaging for 4 days.

### GLUT5 protein and mRNA abundance correlate with fructolytic ability

We cultured the parental and semi-trained PC3 cells for either 24 or 48 h in media containing either 11 mM glucose (full RPMI) or 1 mM glucose plus 10 mM fructose (Figure S4A). We then extracted RNA and performed next-generation sequencing to analyze expression across the transcriptome (RNA-seq) to capture intrinsic differences between the cells. Small differences in gene expression between the parental and semi-trained cells would presumably be enhanced in the trained cells.

**Fig. 3:**
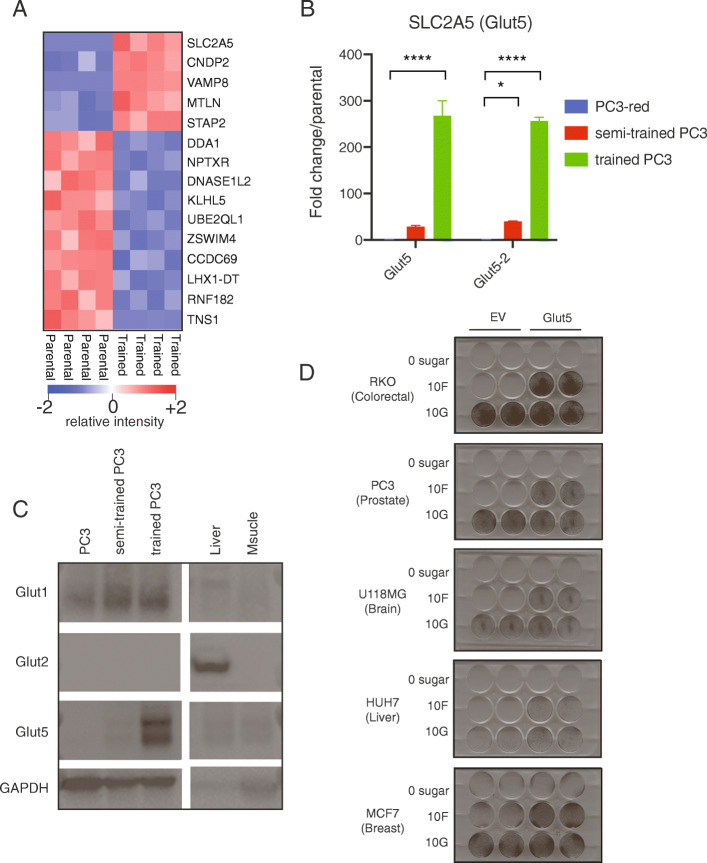
GLUT5 overexpression rescues cellular proliferation in fructose. **a** Normalized expression of genes that are differentially expressed (*q* = 0.4, >1.1 log_2_ fold change) between PC3 and semi-trained PC3 cells (passage 20) presented in heatmap form. **b** Relative expression of *SLC2A5* transcript in semi-trained PC3 and trained PC3 cells as compared to the parental PC3 line. Two primer sets were used (*n* = 2 per condition). Two-way ANOVA with Fisher’s LDS test. *P < 0.05, ****P < 0.0001. **c** Immunoblot of the indicated proteins using lysates from PC3, semi-trained PC3, and trained PC3 cells. The murine liver and muscle used as controls. **d** GLUT5 or an empty vector (EV) were overexpressed in the indicated cells lines. The cells were plated at 20,000-30,000 cells/well and then grown in the presence of no sugar, 10 mM fructose, or 10 mM glucose. After 3 days, the cells were fixed and stained with crystal violet solution (*n* = 2 per condition)

The RNA-seq results were first summarized in a 3-dimensional principal components analysis (PCA), which revealed unique clusters separating the parental from semi-trained cells as well as the different media conditions (Figure S4B). Only fifteen genes were differentially expressed between the parental and semi-trained cells, even when using a generous statistical threshold (*q*=0.4 and log_2_ fold change >1.1), confirming that the cells remained very similar despite being separated for > 20 passages (Fig. 3a, Figure S4C). We validated the expression of these 15 genes together with several fructolytic and glycolytic enzymes using cDNA from parental, semi-trained, and trained cells (Fig. 3b, S4D, S4F). From these data, we observed that the expression of *SLC2A5* had the highest fold change difference and correlated with fructolytic ability. There was a >30× fold increase in semi-trained cells and >200× increase in trained PC3 cells (Fig. 3b). GLUT5 protein levels were also increased in trained PC3 cells compared to their parental PC3 cells (Fig. 3c). We further showed that the increased level of GLUT5 expression was not due to an increase in *SLC2A5* copy number (Figure S4E).

### GLUT5 overexpression rescues growth with fructose across cell lines of different origin independent of KHK

To test whether GLUT5 permits fructolytic growth in other cell lines, we overexpressed GLUT5 in the brain, breast, prostate, colon, and liver cancer cell lines and repeated the proliferation assays. The overexpression of GLUT5 was sufficient to permit cellular proliferation in fructose without affecting expression of other fructolytic or glycolytic genes (Fig. 3d, Figure S5A-C). However, this proliferation required at least 5 mM fructose in the media (Figure S6A-D). We quantified the fructose-mediated proliferation at 96 h and found that fructose contributed significantly to proliferation in the trained and GLUT5-overexpressing cells only in the absence of glucose (Figure S6E-F). These data suggest that the proliferative contributions of glucose and fructose are through metabolism by a common molecular enzyme that preferentially binds glucose.

KHK has been described as a rate-limiting enzyme for fructose metabolism in tumor and normal tissue [9, 15, 37]. To test whether KHK overexpression rescues fructose-mediated cell growth, we overexpressed KHK-A in non-fructolytic RKO cells and saw no rescue of cell proliferation (Figure S7A-B).

### GLUT5 overexpression increases fructose flux into glycolysis

**Fig. 4:**
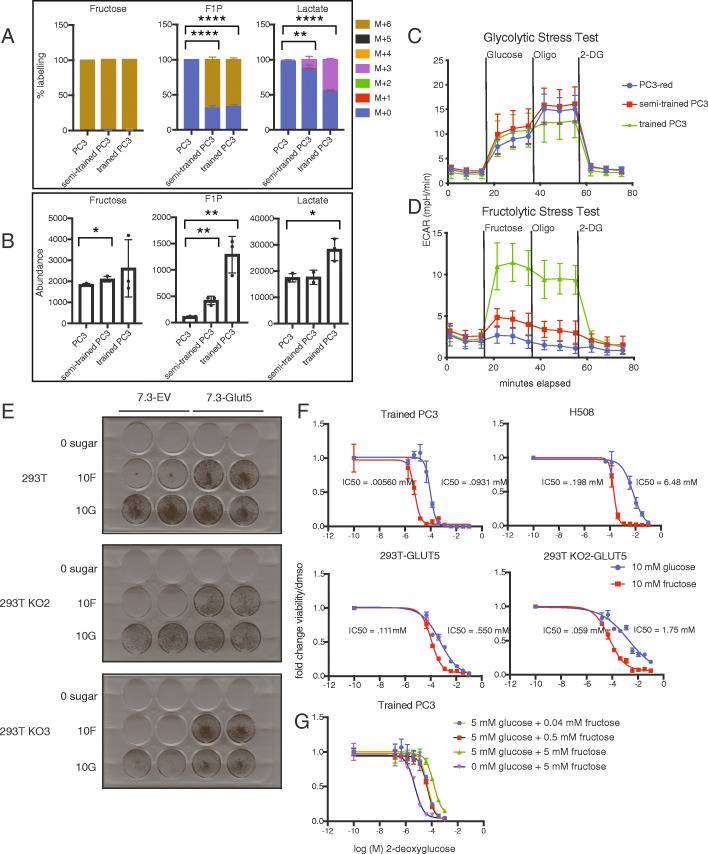
Fructose fluxes through HK, not KHK, in order to sustain cellular proliferation. a Percent of heavy isotope (^13^C) incorporation into fructose, fructose 1-phosphate (F1P), and lactate as detected by LC/MS from polar extracts of PC3, semi-trained PC3, and trained PC3 cells (*n* = 2-3). The isotopic labelling is indicated by M+# designation indicated in the legend where the # represents the amount of [^12^C] replaced by [^13^C]. Two-tailed unpaired *t* tests were used between parental and trained cells (M+3 for lactate, M+6 for fructose/F1P). *P < 0.05, **P< 0.01, and ****P < 0.0001. **b** Total abundance of fructose, F1P, and lactate as detected by LC/MS from polar extracts of PC3, semi-trained PC3, and trained PC3 cells (*n* = 2–3). Two-tailed unpaired *t* tests were used between parental and trained cells. *P < 0.05, **P< 0.01, and ****P < 0.0001. **c** Extracellular acidification rate (ECAR) over time of PC3, semi-trained PC3, and trained PC3 cells under basal conditions and following the addition of glucose, oligomycin (Oligo), and 2-deoxyglucose (2-DG) at the indicated times. Data are the mean and SEM from 6 replicates. **d** ECAR over time of PC3, semi-trained PC3, and trained PC3 cells under basal conditions and following the addition of fructose, Oligo, and 2-DG at the indicated times. Data are the mean and SEM from 6 replicates. **e** GLUT5 or an empty vector (EV) were overexpressed in 293T or 293T *KHK*^-/-^ cells. The cells were plated at 20,000 cells/well and then grown in the presence of no sugar, 10 mM fructose, or 10 mM glucose. After 7 days, the cells were fixed, stained with crystal violet solution (*n* = 2 per condition). **f** Fold change in cell viability as assessed by ATP concentration (Cell Titer Glo) of the indicated fructolytic cell lines grown in either 10 mM glucose or 10 mM fructose containing the specified concentrations of 2-DG after 72 h (*n* = 3 per concentration). The half maximal inhibitory concentration (IC_50_) is displayed on the graph for each curve. **g** Fold change in cell viability as assessed by ATP concentration (Cell Titer Glo) of the trained PC3 grown in the specified sugar conditions containing the specified concentrations of 2-DG after 96 h. (*n* = 2 per concentration). The half maximal inhibitory concentration (IC_50_) is displayed on the graph for each curve

To measure differences in fructose metabolism between non-fructolytic and fructolytic cells, we cultured parental, semi-trained, and trained cells in media containing 10 mM [U-^13^C]-fructose and traced its metabolic fate. The trained cells demonstrated increased levels of fructose-derived carbon into F1P, lactate, and TCA cycle intermediates (Fig. 4a, b, Figure S8A-B). Measurable amounts of fructose were also detected in PC3 cells, suggesting that fructose can be imported into cells but does not meet the concentration necessary to sustain proliferation.

In order to gain real-time insight into the ability of fructose to acidify the media (presumably via lactate production), we measured the extracellular acidification rate (ECAR) using parental and trained PC3 cells (Fig. 4c,d). While both cell types had similar ECAR in response to glucose, trained cells had much higher ECAR in response to fructose. Semi-trained cells showed an intermediate phenotype, as expected. Interestingly, 2-deoxyglucose (2-DG), a competitive inhibitor for HK, immediately extinguished both glucose- and fructose-induced ECAR. This fact led us to hypothesize that fructose flux to lactate is mediated by HK rather than the canonical fructose-metabolism protein, KHK.

### Cells proliferate with fructose through hexokinase

Using CRISPR-Cas9, we generated a *KHK*^*-/-*^ line using 293T cells (293T *KHK*^-/-^) (Figure S9A-B). We then overexpressed either an empty vector (EV) or V5-tagged GLUT5 in the parental and *KHK*^-/-^ cells (Figure S9C). The resulting cells were cultured in 10 mM [U-^13^C]-fructose prior to recovery of polar metabolites for metabolomics. GLUT5 overexpression greatly increased the abundance of F1P and its proportion of fructose-derived carbons in the parental but not the *KHK*^-/-^ cells (Figure S9D-E). However, the abundance and isotopic labeling patterns of lactate and TCA cycle intermediates were similar between GLUT5-overexpressing parental and *KHK*^-/-^ cells (Figure S9D-E). Moreover, the absence of F1P did not affect cellular proliferation with fructose, as GLUT5 overexpression rescued fructose-mediated proliferation in both the parental as well as the *KHK*^-/-^ cells (Fig. 4e). We therefore conclude that KHK is dispensable for fructose-mediated cell proliferation.

We capitalized on the kinetic properties of HK to discern whether fructose-mediated cell proliferation was mediated by KHK or HK. HK has a higher affinity for glucose than it does for fructose [38]. Therefore, we hypothesized that if cells used KHK for growth, then they would be more resistant to the HK inhibitor, 2-DG, when cultured in fructose as compared to glucose. Alternatively, we hypothesized that if cells primarily used HK for growth, then they would be more sensitive to 2-DG when cultured in fructose as compared to glucose. We treated cells with increasing concentrations of 2-DG in media containing either 10 mM fructose or 10 mM glucose and found that cells in the fructose media were 5–33× more sensitive to 2-DG (Fig. 4f). At lower levels of sugar (5 mM), the fructose-treated cells remain more sensitive to 2-DG than glucose-treated cells; however, this effect is lost when the sugars are given together (Fig. 4g). Therefore, we conclude that cells can adapt to metabolize fructose through upregulation of GLUT5 and metabolism through HK instead of KHK.

## Discussion

Cells preferentially metabolize the nutrients available in their microenvironment. Transformed cells acquire the ability to metabolize novel nutrients which allow them to outgrow their neighbors and survive in sites of metastasis. Understanding how tumor cells acquire this ability is valuable given the growing interest in metabolic and dietary interventions as anti-cancer therapy [39]. Here, we show that human cancer cells from a wide range of origins can acquire the ability to metabolize fructose simply by stable overexpression of GLUT5. These data suggest that sugar uptake can be a limiting factor preventing fructose-mediated cell proliferation.

Sugar uptake is also a key regulatory node for glucose metabolism and growth. For example, the expression of the glucose transporters, GLUT1 and GLUT4, control skeletal muscle glucose uptake at rest, and in response to contraction or insulin [40, 41]. Additionally, the expression of GLUT1 and GLUT3 in tumors is associated with enhanced glucose uptake and oncogenic growth [42–44]. Tumor cells continue to regulate the flux of glucose at the levels of phosphorylation by HK, fructose-1,6-bisphosphate production by phosphofructokinase, and lactate export [45]. In this study, we show that fructose phosphorylation by KHK is not required for fructose metabolism and cell growth; however, we speculate that other regulatory nodes exist.

Our conclusions are supported by clinical evidence from subjects with cancer. GLUT5 is significantly upregulated in tumors from patients with the colon, lung, and breast adenocarcinoma, acute myeloid leukemia, ovarian carcinoma, and glioma where it contributes to malignancy and poor survival [16, 21, 23, 25–27]. Many of these studies investigated fructose metabolism in the absence of glucose, using a wide range of fructose concentrations (ref 22: 6 mM, ref 24: 25 mM, ref 26: 1.5-6 mM, ref 27: 3 mM, ref 28: 6 mM). It is worth noting that these studies were able to discern physiologically relevant findings despite modelling fructose-mediated growth in the absence of glucose in vitro.

Our data confirms that GLUT5 overexpression is sufficient to promote cellular proliferation in fructose, but the abundance of the GLUT5 transcript in our initial profiling did not predict the fructolytic index across cell lines. For example, H508 (fructolytic) and RKO (non-fructolytic) cells are from the same colorectal origin with similar levels of GLUT5 yet have vastly different abilities to proliferate in fructose. Other groups have shown that the stability of GLUT5 mRNA and the location of GLUT5 protein can be modulated by distinct signaling pathways [5, 46]. Therefore, we conclude that GLUT5 expression needs to be analyzed in tandem with other, currently unknown, cellular features in order to determine fructose-mediated proliferation a priori.

Our data supports the conclusion that GLUT5 is a robust determinant of fructose-mediated cell proliferation. However, we were unable to identify how the semi-trained and trained cells upregulated this message. There was no difference in *SLC2A5* copy number in the genomic DNA and minimal change in the expression of known *SLC2A5*-regulating fructose-response elements like Chrebpβ (Fig. 3, Figure S3). Due to the specificity of the *SLC2A5* overexpression, we hypothesize that the upregulation stems from epigenetic or genetic modifications at the *SLC2A5* locus.

Our data suggest that KHK is dispensable for fructose-mediated proliferation. Instead, we show that cancer cells metabolize fructose using HK, as is the case in lower order organisms. For example, *Hk* is the only fructokinase in yeast and the flux through HK sustains the high activity of nectarivore flight muscles [47, 48]. In humans, fructose is thought to be primarily metabolized by KHK, but this may be unique to non-proliferative cells in the liver, intestine, and kidney. Proliferating cells typically switch to less fructolytic isoforms of KHK. For example, liver cancer cells convert from the high affinity KHK-c variant (Km = 0.7 mM), to the low affinity isoform, KHK-a (Km = 7 mM), that may play a role in de novo nucleotide biosynthesis [49]. On average, the cell lines we profiled in this study expressed >160× more KHK-a than KHK-c (Fig. 1e, Table S1). Furthermore, the expression of HK (Km for fructose 1–4 mM) is greater than KHK-a in these cells, which may explain the preference for this route of metabolism [38, 50]. Our data suggests that this route is most relevant in tissues such as the liver, kidney, seminal vesicles, and prostate, where fructose levels achieve concentrations higher than 5 mM [17, 51, 52].

The exact role of KHK and F1P in these cell lines remain unclear. KHK-mediated fructose metabolism may become more important when HK is saturated or inhibited by high concentrations of glucose and glucose 6-phosphate. However, it is unclear if glucose ever reaches these high concentrations in poorly vascularized solid tumors [53]. For example, pancreatic adenocarcinomas in mice have significantly less glucose in the tumor interstitial fluid relative to plasma [54]. These poorly vascularized tumors also receive less oxygen from the blood [53], and the resulting hypoxia enhances the endogenous production of fructose and the expression of fructolytic genes [9, 55–58].

In conclusion, our study defines fructose uptake as a limiting factor for fructose-mediated cell proliferation. We describe a previously unappreciated role of HK to permit fructolytic cell growth. These findings advance our basic understanding of fructose metabolism in cancer cells and highlight a limitation of directly targeting KHK for anti-cancer therapy.

## Conclusions

The intent of this study was to find the determinants of fructose-mediated proliferation in cell lines. We have found that fructose-dependent proliferation of cancer cells is not determined by tissue of origin nor expression of any individual fructolytic gene. Using a positive selection approach, we were able to train PC3 cells to proliferate with fructose. We saw that GLUT5 was strongly upregulated in trained cells and that overexpressing GLUT5 allowed non-fructolytic cell lines of several different origins to proliferate in fructose. Lastly, we showed that cells metabolize fructose through hexokinase, not ketohexokinase, to sustain proliferation and glycolysis. This study sheds light on cell-autonomous fructose metabolism and suggests that targeting fructose metabolism may require inhibition of both KHK as well as HK.

## Supplementary Information

**Additional file 1: Supplemental Figure 1.** Cell growth in fructose is heterogeneous. **Supplemental Figure 2.** Gene expression does not determine the fructolytic index. **Supplemental Figure 3.** Cells can stably utilize fructose for proliferation. **Supplemental Figure 4.** *SLC2A5* copy number, validated RNA-seq transcripts (excluding *SLC2A5*), and selected metabolic enzyme transcripts do not correlate with fructolytic ability. **Supplemental Figure 5.** Selected metabolism genes are not changed with GLUT5 overexpression. **Supplemental Figure 6.** Serum concentration of glucose overshadows fructose contributions to proliferation rate. **Supplemental Figure 7.** KHK overexpression does not rescue the ability to proliferate in fructose. **Supplemental Figure 8.** Trained PC3 have increased fructose flux into the TCA cycle. **Supplemental Figure 9.** Trained PC3 have increased fructose flux into the TCA cycle. **Supplemental Table 1**: Clinical and genomic data of profiled cell lines in order of fructolytic index. Related to Figure 1. **Supplemental Table 2.** qPCR data for each cell line using primers from Supplemental File 1. (*n =* 2 per gene per sample, 2^dC_t_ values shown). Related to Figure 1. **Supplemental Table 3.** qPCR primers for selected metabolic genes, CRISPR-cas9 primers, and qPCR primers for gDNA. Related to Figures 1, 4 and Supplemental Figures 4, 8. **Supplemental Table 4.** qPCR primers for RNA-seq hits, related to Figure 3 and Supplemental Figure 4.

## Abbreviations

HK: Hexokinase
KHK: Ketohexokinase
ALDOB: Aldolase B
F1P: Fructose-1-phosphate
FBS: Fetal bovine serum
dFBS: Dialyzed FBS
PCA: Principle components analysis
2-DG: 2-Deoxyglucose
EV: Empty vector
SEM: Standard error of the mean
