# Supplementary figures and images for: GLUT5 (SLC2A5) enables fructose-mediated proliferation independent of ketohexokinase
Source: Cancer Metab. 2021 Mar 24;9:12. doi: 10.1186/s40170-021-00246-9 (PMC7992954; doi:10.1186/s40170-021-00246-9)

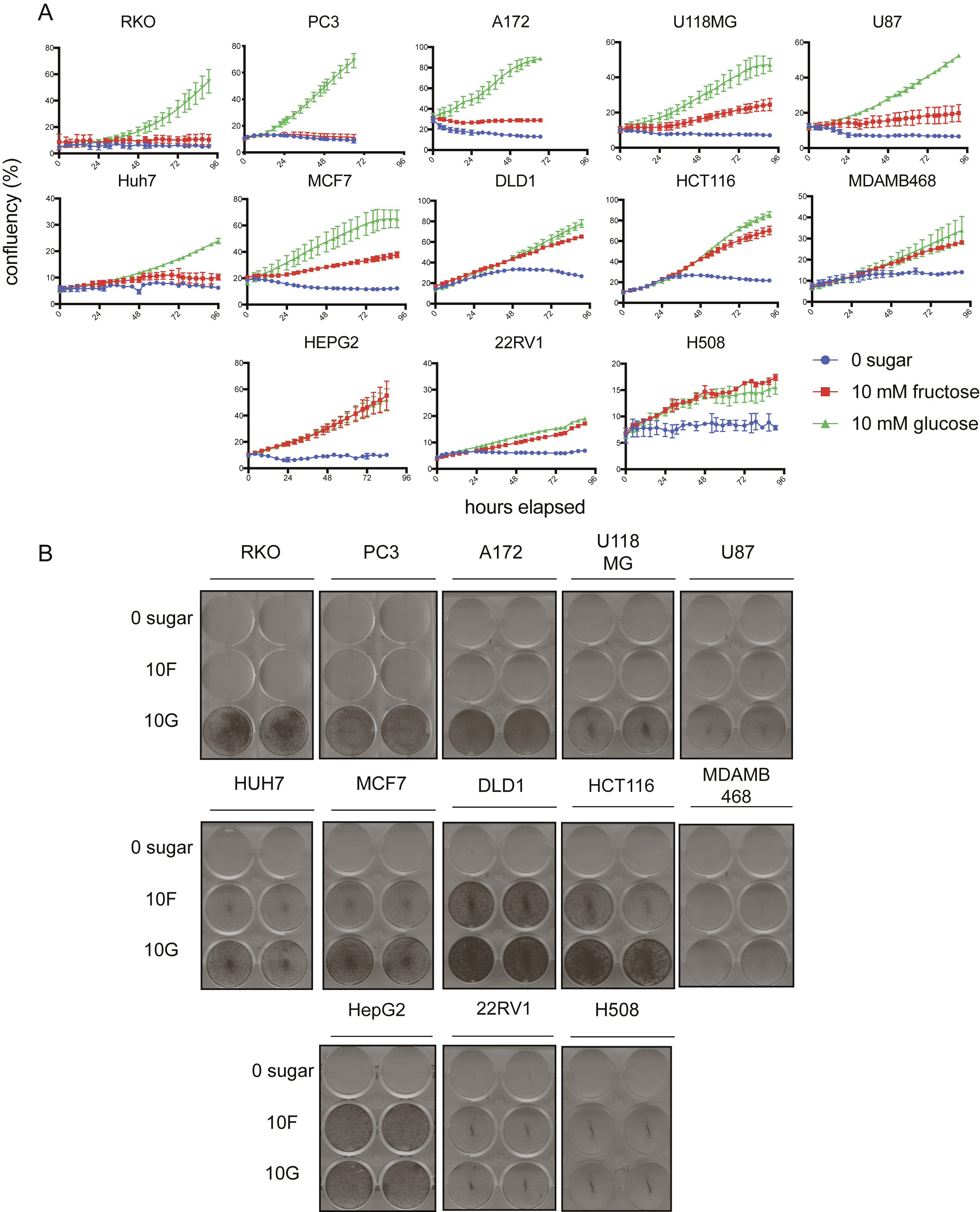

Supplement: Supplementary file 3 — Additional file 1: Supplemental Figure 1. Cell growth in fructose is heterogeneous. Supplemental Figure 2. Gene expression does not determine the fructolytic index. Supplemental Figure 3. Cells can stably utilize fructose for proliferation. Supplemental Figure 4. SLC2A5 copy number, validated RNA-seq transcripts (excluding SLC2A5), and selected metabolic enzyme transcripts do not correlate with fructolytic ability. Supplemental Figure 5. Selected metabolism genes are not changed with GLUT5 overexpression. Supplemental Figure 6. Serum concentration of glucose overshadows fructose contributions to proliferation rate. Supplemental Figure 7. KHK overexpression does not rescue the ability to proliferate in fructose. Supplemental Figure 8. Trained PC3 have increased fructose flux into the TCA cycle. Supplemental Figure 9. Trained PC3 have increased fructose flux into the TCA cycle. Supplemental Table 1: Clinical and genomic data of profiled cell lines in order of fructolytic index. Related to Figure 1. Supplemental Table 2. qPCR data for each cell line using primers from Supplemental File 1. (n = 2 per gene per sample, 2^dCt values shown). Related to Figure 1. Supplemental Table 3. qPCR primers for selected metabolic genes, CRISPR-cas9 primers, and qPCR primers for gDNA. Related to Figures 1, 4 and Supplemental Figures 4, 8. Supplemental Table 4. qPCR primers for RNA-seq hits, related to Figure 3 and Supplemental Figure 4. [file 40170_2021_246_MOESM1_ESM.zip › SUPPLEMENTAL 1.tif]

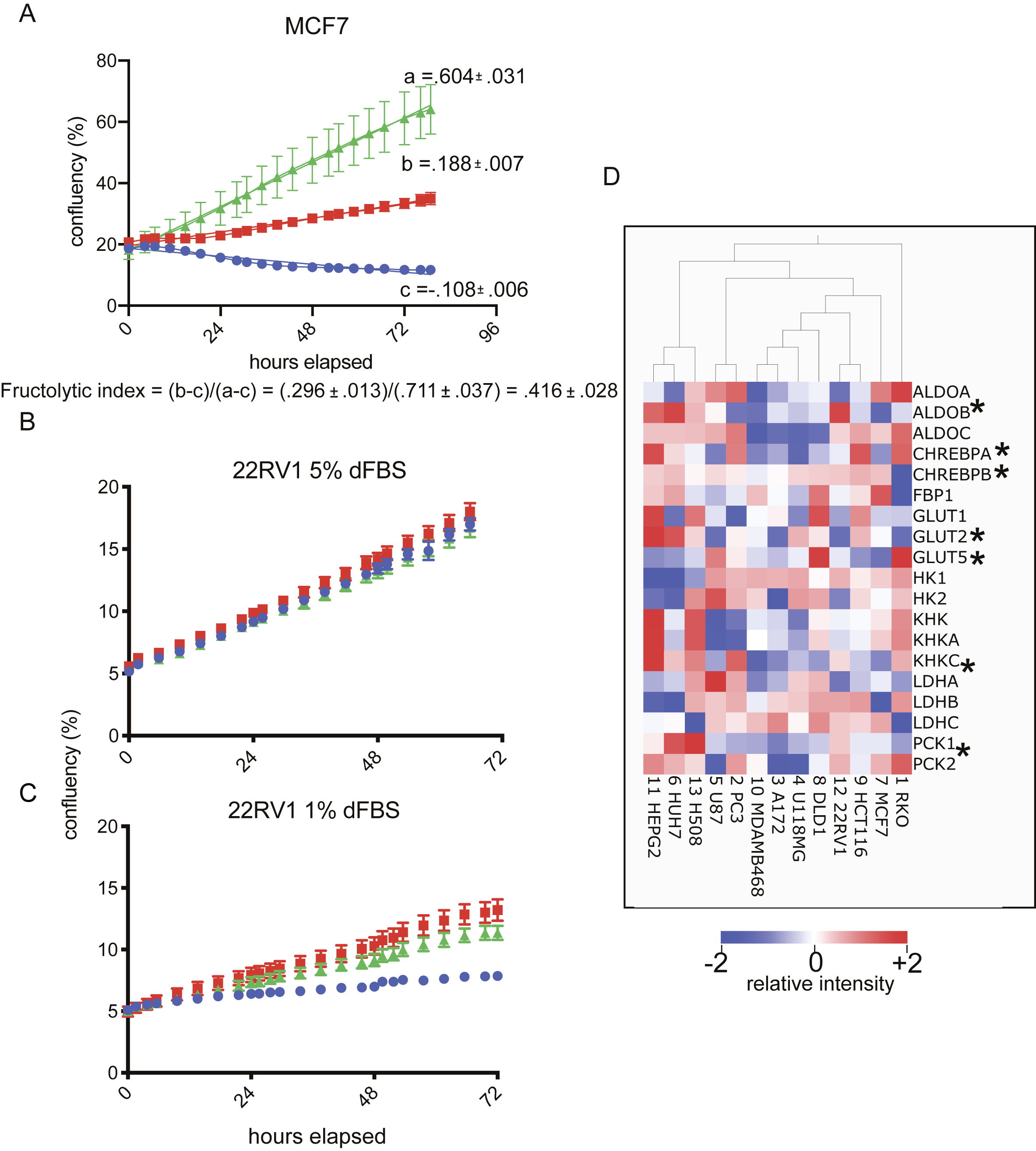

Supplement: Supplementary file 3 — Additional file 1: Supplemental Figure 1. Cell growth in fructose is heterogeneous. Supplemental Figure 2. Gene expression does not determine the fructolytic index. Supplemental Figure 3. Cells can stably utilize fructose for proliferation. Supplemental Figure 4. SLC2A5 copy number, validated RNA-seq transcripts (excluding SLC2A5), and selected metabolic enzyme transcripts do not correlate with fructolytic ability. Supplemental Figure 5. Selected metabolism genes are not changed with GLUT5 overexpression. Supplemental Figure 6. Serum concentration of glucose overshadows fructose contributions to proliferation rate. Supplemental Figure 7. KHK overexpression does not rescue the ability to proliferate in fructose. Supplemental Figure 8. Trained PC3 have increased fructose flux into the TCA cycle. Supplemental Figure 9. Trained PC3 have increased fructose flux into the TCA cycle. Supplemental Table 1: Clinical and genomic data of profiled cell lines in order of fructolytic index. Related to Figure 1. Supplemental Table 2. qPCR data for each cell line using primers from Supplemental File 1. (n = 2 per gene per sample, 2^dCt values shown). Related to Figure 1. Supplemental Table 3. qPCR primers for selected metabolic genes, CRISPR-cas9 primers, and qPCR primers for gDNA. Related to Figures 1, 4 and Supplemental Figures 4, 8. Supplemental Table 4. qPCR primers for RNA-seq hits, related to Figure 3 and Supplemental Figure 4. [file 40170_2021_246_MOESM1_ESM.zip › SUPPLEMENTAL 2.tif]

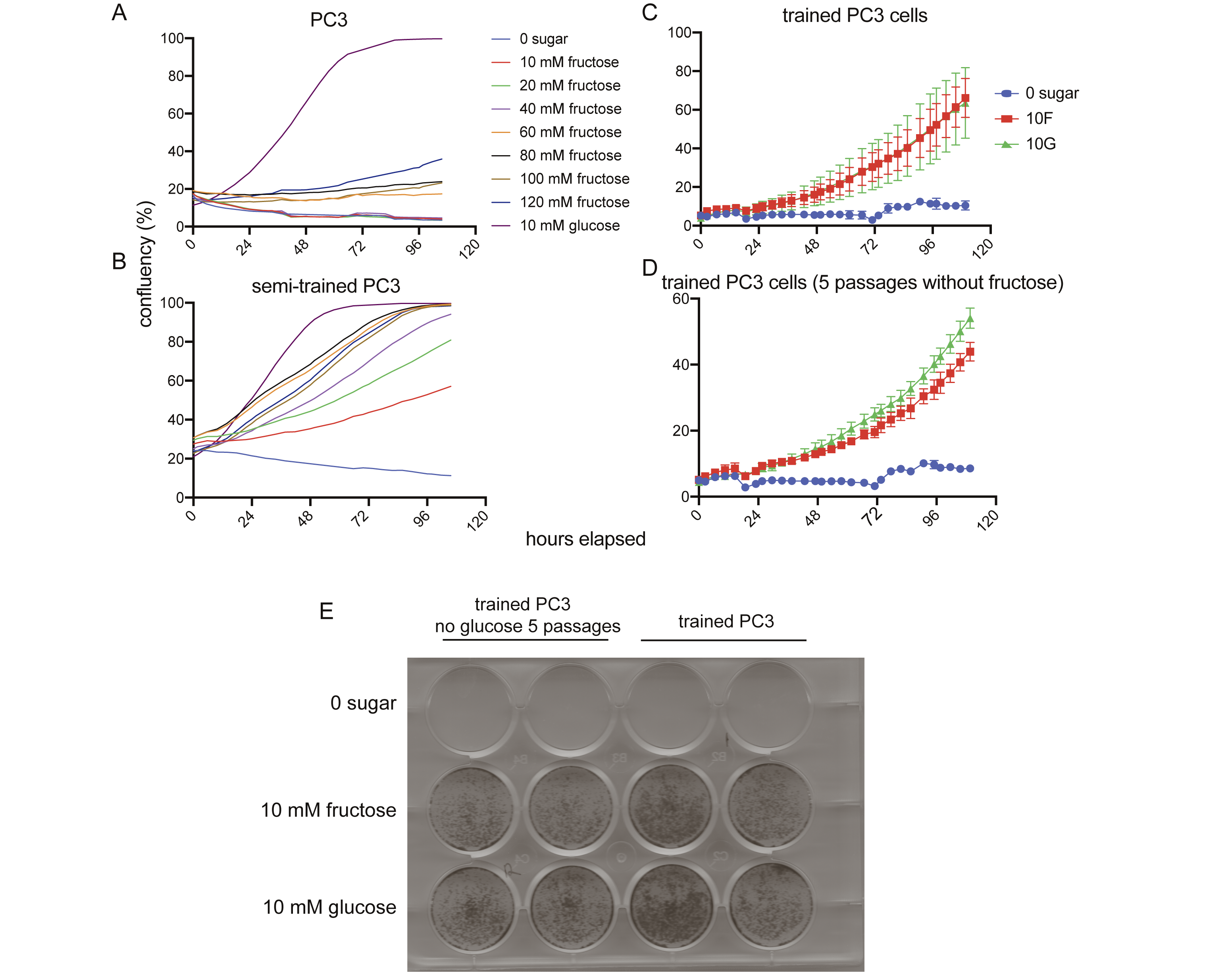

Supplement: Supplementary file 3 — Additional file 1: Supplemental Figure 1. Cell growth in fructose is heterogeneous. Supplemental Figure 2. Gene expression does not determine the fructolytic index. Supplemental Figure 3. Cells can stably utilize fructose for proliferation. Supplemental Figure 4. SLC2A5 copy number, validated RNA-seq transcripts (excluding SLC2A5), and selected metabolic enzyme transcripts do not correlate with fructolytic ability. Supplemental Figure 5. Selected metabolism genes are not changed with GLUT5 overexpression. Supplemental Figure 6. Serum concentration of glucose overshadows fructose contributions to proliferation rate. Supplemental Figure 7. KHK overexpression does not rescue the ability to proliferate in fructose. Supplemental Figure 8. Trained PC3 have increased fructose flux into the TCA cycle. Supplemental Figure 9. Trained PC3 have increased fructose flux into the TCA cycle. Supplemental Table 1: Clinical and genomic data of profiled cell lines in order of fructolytic index. Related to Figure 1. Supplemental Table 2. qPCR data for each cell line using primers from Supplemental File 1. (n = 2 per gene per sample, 2^dCt values shown). Related to Figure 1. Supplemental Table 3. qPCR primers for selected metabolic genes, CRISPR-cas9 primers, and qPCR primers for gDNA. Related to Figures 1, 4 and Supplemental Figures 4, 8. Supplemental Table 4. qPCR primers for RNA-seq hits, related to Figure 3 and Supplemental Figure 4. [file 40170_2021_246_MOESM1_ESM.zip › SUPPLEMENTAL 3.tif]

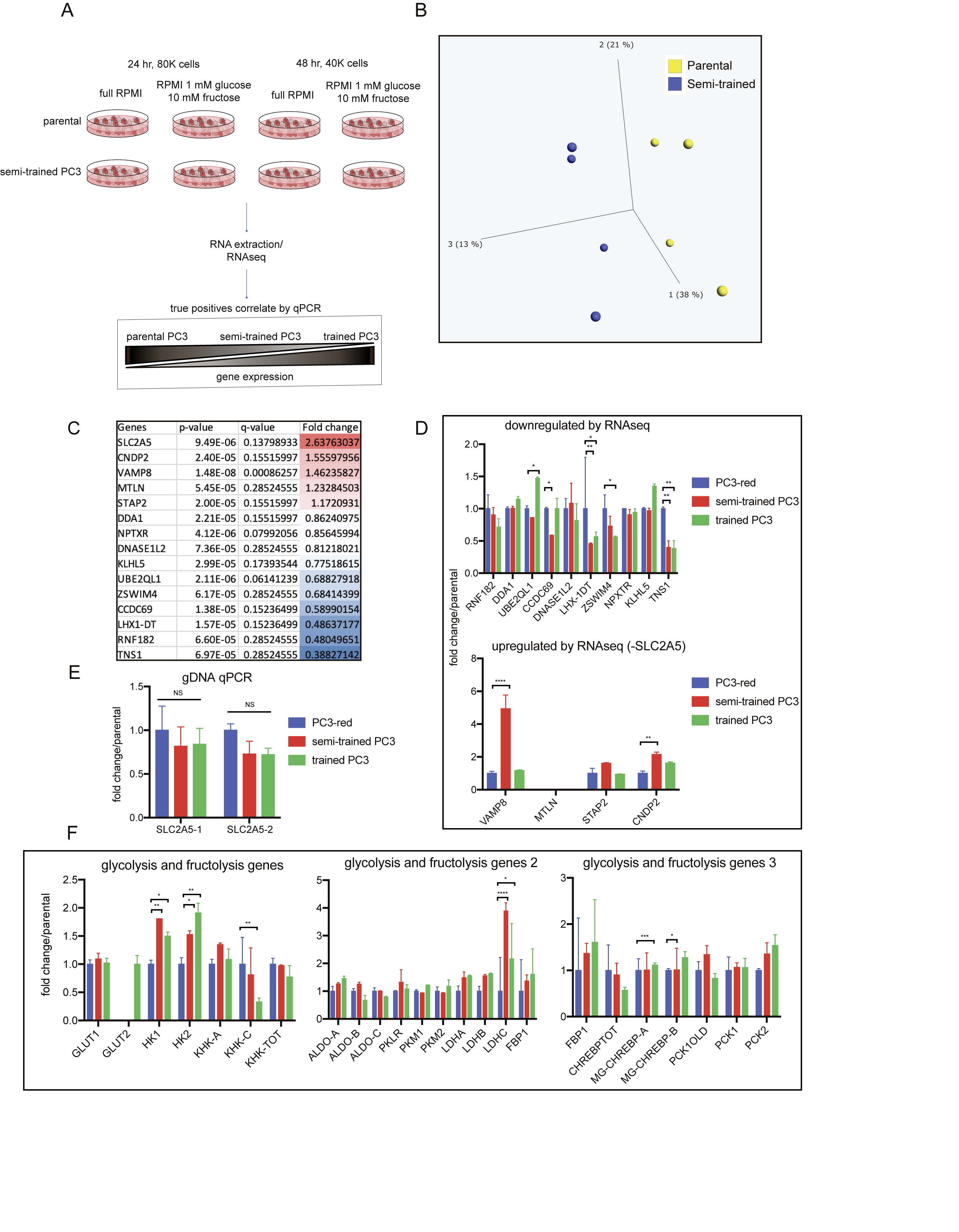

Supplement: Supplementary file 3 — Additional file 1: Supplemental Figure 1. Cell growth in fructose is heterogeneous. Supplemental Figure 2. Gene expression does not determine the fructolytic index. Supplemental Figure 3. Cells can stably utilize fructose for proliferation. Supplemental Figure 4. SLC2A5 copy number, validated RNA-seq transcripts (excluding SLC2A5), and selected metabolic enzyme transcripts do not correlate with fructolytic ability. Supplemental Figure 5. Selected metabolism genes are not changed with GLUT5 overexpression. Supplemental Figure 6. Serum concentration of glucose overshadows fructose contributions to proliferation rate. Supplemental Figure 7. KHK overexpression does not rescue the ability to proliferate in fructose. Supplemental Figure 8. Trained PC3 have increased fructose flux into the TCA cycle. Supplemental Figure 9. Trained PC3 have increased fructose flux into the TCA cycle. Supplemental Table 1: Clinical and genomic data of profiled cell lines in order of fructolytic index. Related to Figure 1. Supplemental Table 2. qPCR data for each cell line using primers from Supplemental File 1. (n = 2 per gene per sample, 2^dCt values shown). Related to Figure 1. Supplemental Table 3. qPCR primers for selected metabolic genes, CRISPR-cas9 primers, and qPCR primers for gDNA. Related to Figures 1, 4 and Supplemental Figures 4, 8. Supplemental Table 4. qPCR primers for RNA-seq hits, related to Figure 3 and Supplemental Figure 4. [file 40170_2021_246_MOESM1_ESM.zip › SUPPLEMENTAL 4.tif]

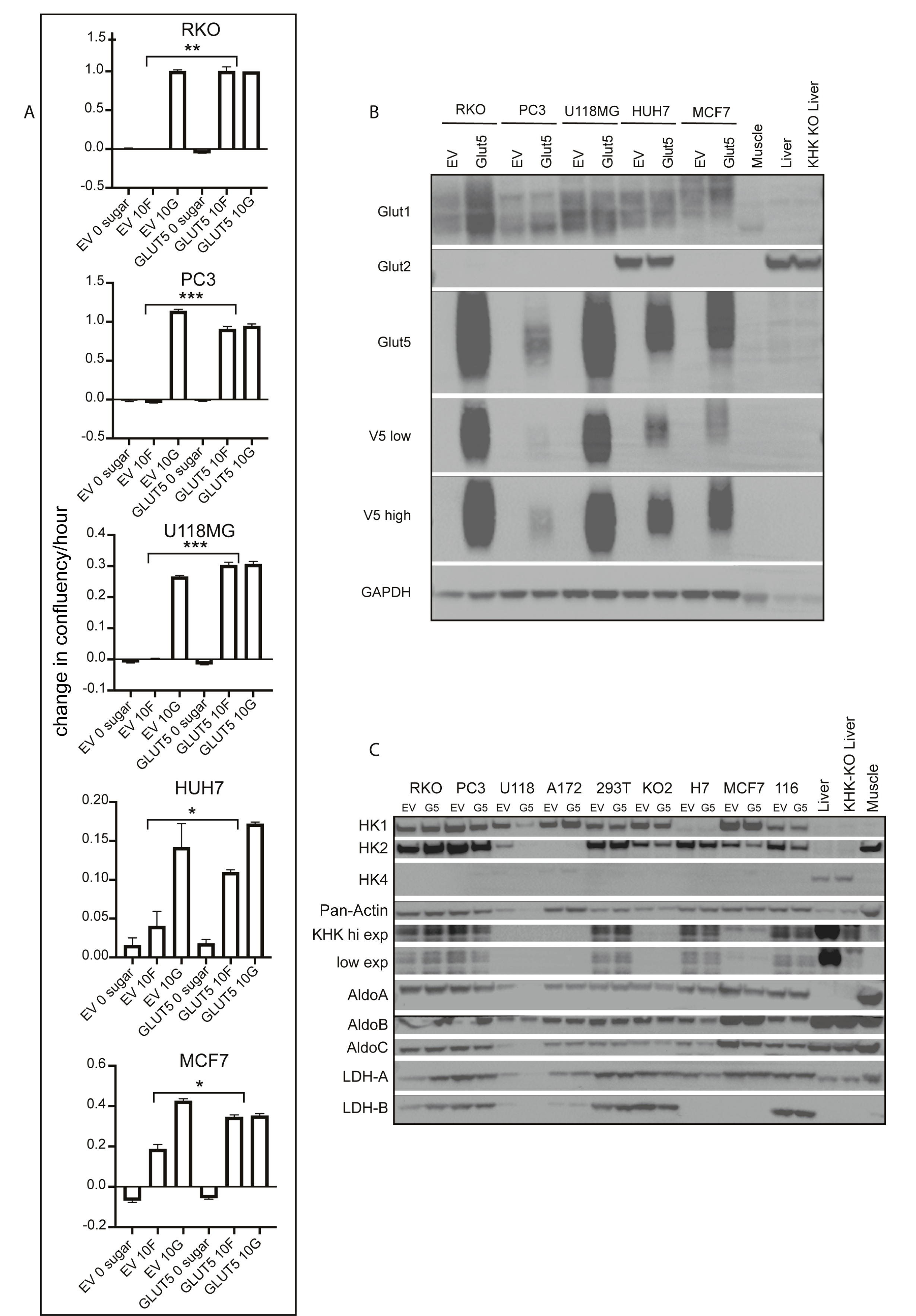

Supplement: Supplementary file 3 — Additional file 1: Supplemental Figure 1. Cell growth in fructose is heterogeneous. Supplemental Figure 2. Gene expression does not determine the fructolytic index. Supplemental Figure 3. Cells can stably utilize fructose for proliferation. Supplemental Figure 4. SLC2A5 copy number, validated RNA-seq transcripts (excluding SLC2A5), and selected metabolic enzyme transcripts do not correlate with fructolytic ability. Supplemental Figure 5. Selected metabolism genes are not changed with GLUT5 overexpression. Supplemental Figure 6. Serum concentration of glucose overshadows fructose contributions to proliferation rate. Supplemental Figure 7. KHK overexpression does not rescue the ability to proliferate in fructose. Supplemental Figure 8. Trained PC3 have increased fructose flux into the TCA cycle. Supplemental Figure 9. Trained PC3 have increased fructose flux into the TCA cycle. Supplemental Table 1: Clinical and genomic data of profiled cell lines in order of fructolytic index. Related to Figure 1. Supplemental Table 2. qPCR data for each cell line using primers from Supplemental File 1. (n = 2 per gene per sample, 2^dCt values shown). Related to Figure 1. Supplemental Table 3. qPCR primers for selected metabolic genes, CRISPR-cas9 primers, and qPCR primers for gDNA. Related to Figures 1, 4 and Supplemental Figures 4, 8. Supplemental Table 4. qPCR primers for RNA-seq hits, related to Figure 3 and Supplemental Figure 4. [file 40170_2021_246_MOESM1_ESM.zip › SUPPLEMENTAL 5.tif]

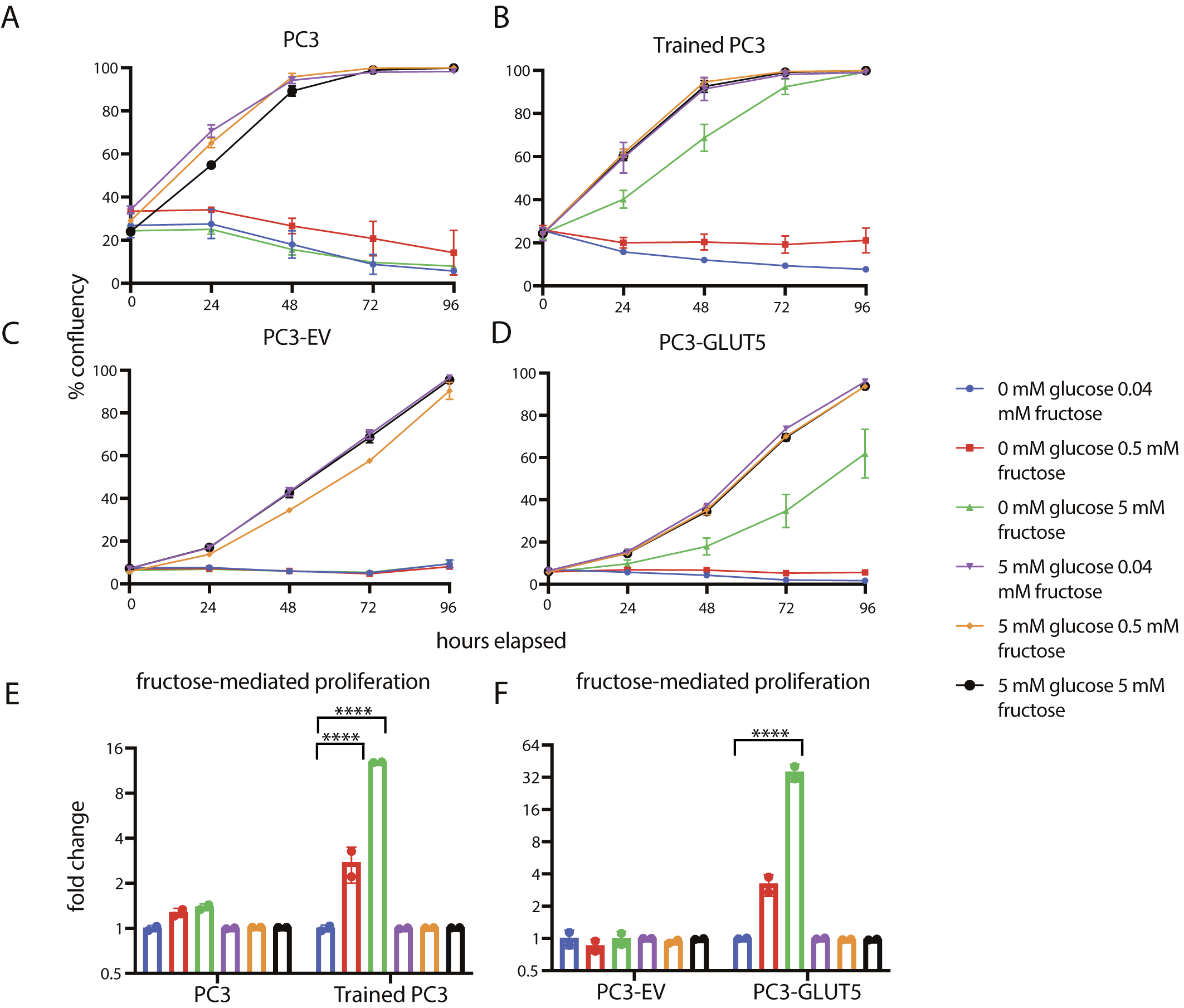

Supplement: Supplementary file 3 — Additional file 1: Supplemental Figure 1. Cell growth in fructose is heterogeneous. Supplemental Figure 2. Gene expression does not determine the fructolytic index. Supplemental Figure 3. Cells can stably utilize fructose for proliferation. Supplemental Figure 4. SLC2A5 copy number, validated RNA-seq transcripts (excluding SLC2A5), and selected metabolic enzyme transcripts do not correlate with fructolytic ability. Supplemental Figure 5. Selected metabolism genes are not changed with GLUT5 overexpression. Supplemental Figure 6. Serum concentration of glucose overshadows fructose contributions to proliferation rate. Supplemental Figure 7. KHK overexpression does not rescue the ability to proliferate in fructose. Supplemental Figure 8. Trained PC3 have increased fructose flux into the TCA cycle. Supplemental Figure 9. Trained PC3 have increased fructose flux into the TCA cycle. Supplemental Table 1: Clinical and genomic data of profiled cell lines in order of fructolytic index. Related to Figure 1. Supplemental Table 2. qPCR data for each cell line using primers from Supplemental File 1. (n = 2 per gene per sample, 2^dCt values shown). Related to Figure 1. Supplemental Table 3. qPCR primers for selected metabolic genes, CRISPR-cas9 primers, and qPCR primers for gDNA. Related to Figures 1, 4 and Supplemental Figures 4, 8. Supplemental Table 4. qPCR primers for RNA-seq hits, related to Figure 3 and Supplemental Figure 4. [file 40170_2021_246_MOESM1_ESM.zip › SUPPLEMENTAL 6.tif]

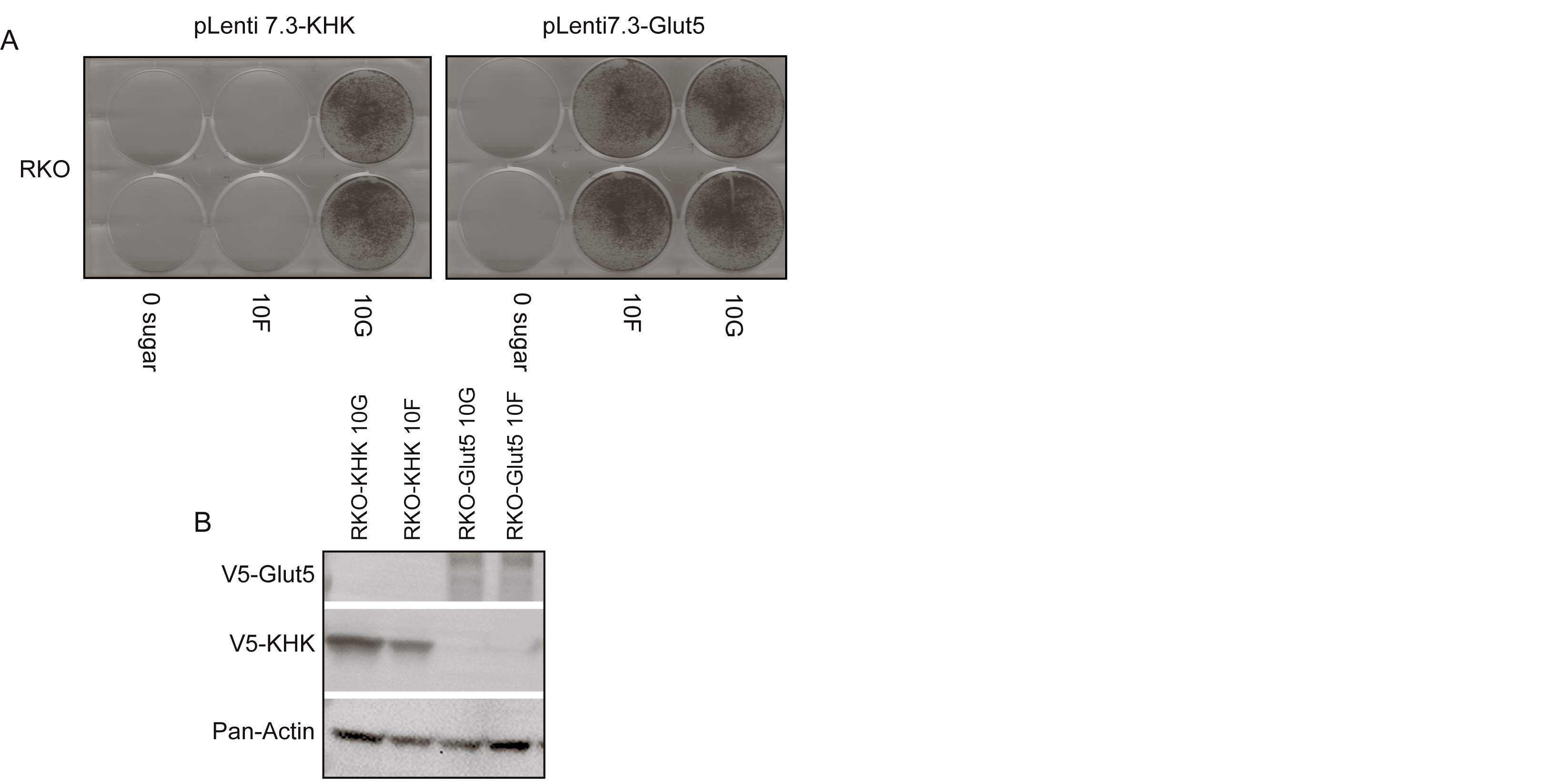

Supplement: Supplementary file 3 — Additional file 1: Supplemental Figure 1. Cell growth in fructose is heterogeneous. Supplemental Figure 2. Gene expression does not determine the fructolytic index. Supplemental Figure 3. Cells can stably utilize fructose for proliferation. Supplemental Figure 4. SLC2A5 copy number, validated RNA-seq transcripts (excluding SLC2A5), and selected metabolic enzyme transcripts do not correlate with fructolytic ability. Supplemental Figure 5. Selected metabolism genes are not changed with GLUT5 overexpression. Supplemental Figure 6. Serum concentration of glucose overshadows fructose contributions to proliferation rate. Supplemental Figure 7. KHK overexpression does not rescue the ability to proliferate in fructose. Supplemental Figure 8. Trained PC3 have increased fructose flux into the TCA cycle. Supplemental Figure 9. Trained PC3 have increased fructose flux into the TCA cycle. Supplemental Table 1: Clinical and genomic data of profiled cell lines in order of fructolytic index. Related to Figure 1. Supplemental Table 2. qPCR data for each cell line using primers from Supplemental File 1. (n = 2 per gene per sample, 2^dCt values shown). Related to Figure 1. Supplemental Table 3. qPCR primers for selected metabolic genes, CRISPR-cas9 primers, and qPCR primers for gDNA. Related to Figures 1, 4 and Supplemental Figures 4, 8. Supplemental Table 4. qPCR primers for RNA-seq hits, related to Figure 3 and Supplemental Figure 4. [file 40170_2021_246_MOESM1_ESM.zip › SUPPLEMENTAL 7.tif]

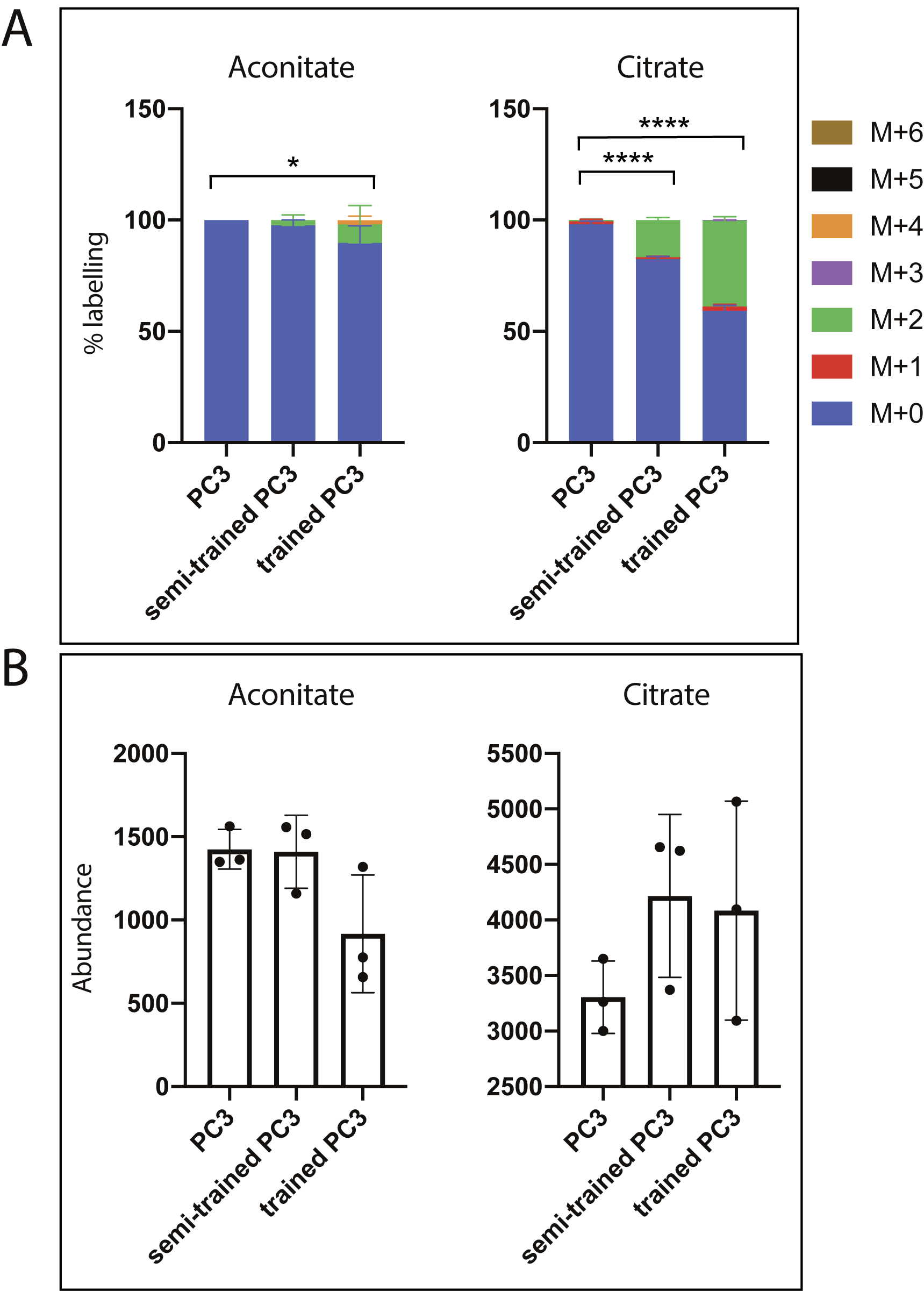

Supplement: Supplementary file 3 — Additional file 1: Supplemental Figure 1. Cell growth in fructose is heterogeneous. Supplemental Figure 2. Gene expression does not determine the fructolytic index. Supplemental Figure 3. Cells can stably utilize fructose for proliferation. Supplemental Figure 4. SLC2A5 copy number, validated RNA-seq transcripts (excluding SLC2A5), and selected metabolic enzyme transcripts do not correlate with fructolytic ability. Supplemental Figure 5. Selected metabolism genes are not changed with GLUT5 overexpression. Supplemental Figure 6. Serum concentration of glucose overshadows fructose contributions to proliferation rate. Supplemental Figure 7. KHK overexpression does not rescue the ability to proliferate in fructose. Supplemental Figure 8. Trained PC3 have increased fructose flux into the TCA cycle. Supplemental Figure 9. Trained PC3 have increased fructose flux into the TCA cycle. Supplemental Table 1: Clinical and genomic data of profiled cell lines in order of fructolytic index. Related to Figure 1. Supplemental Table 2. qPCR data for each cell line using primers from Supplemental File 1. (n = 2 per gene per sample, 2^dCt values shown). Related to Figure 1. Supplemental Table 3. qPCR primers for selected metabolic genes, CRISPR-cas9 primers, and qPCR primers for gDNA. Related to Figures 1, 4 and Supplemental Figures 4, 8. Supplemental Table 4. qPCR primers for RNA-seq hits, related to Figure 3 and Supplemental Figure 4. [file 40170_2021_246_MOESM1_ESM.zip › SUPPLEMENTAL 8.tif]

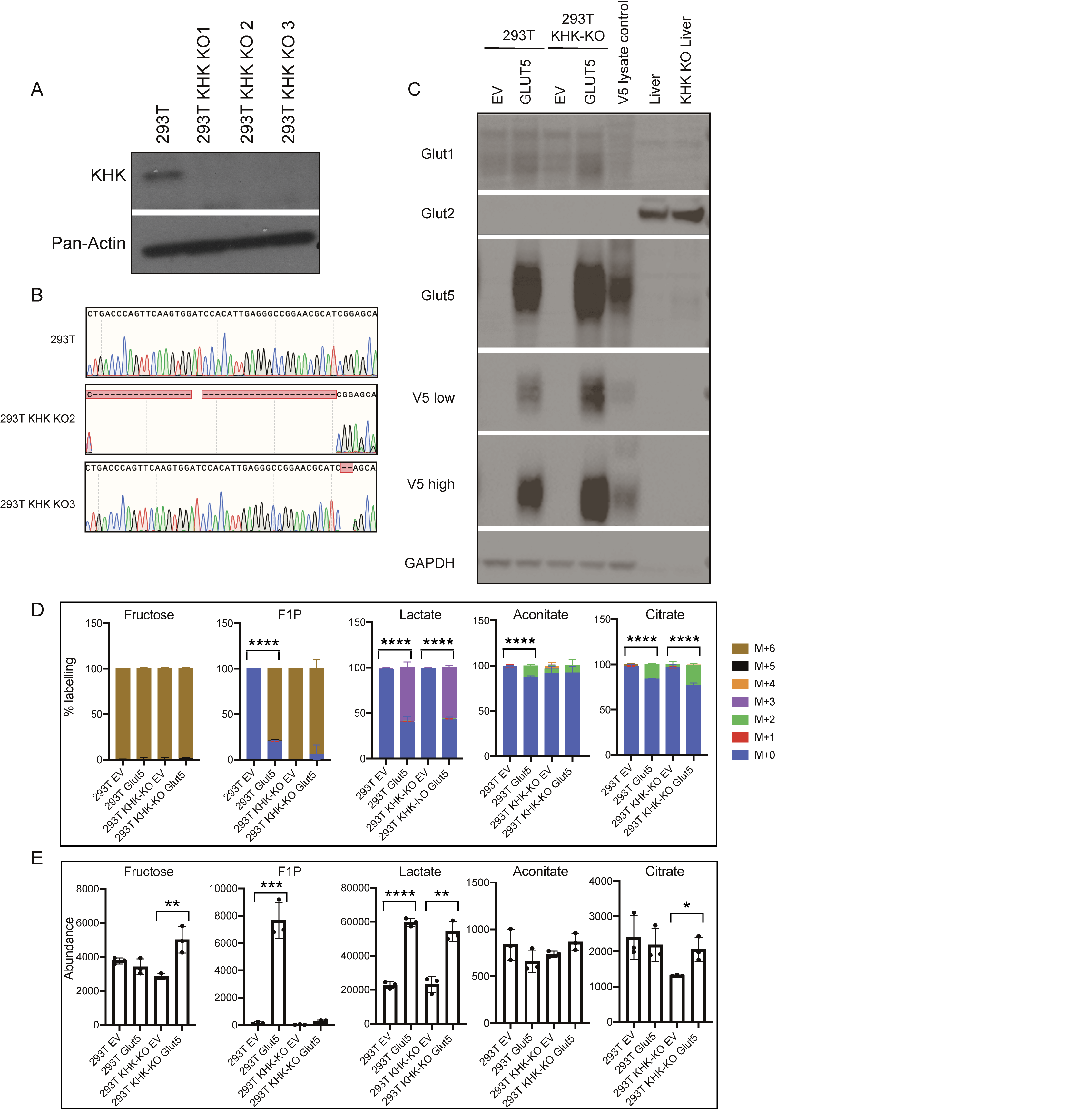

Supplement: Supplementary file 3 — Additional file 1: Supplemental Figure 1. Cell growth in fructose is heterogeneous. Supplemental Figure 2. Gene expression does not determine the fructolytic index. Supplemental Figure 3. Cells can stably utilize fructose for proliferation. Supplemental Figure 4. SLC2A5 copy number, validated RNA-seq transcripts (excluding SLC2A5), and selected metabolic enzyme transcripts do not correlate with fructolytic ability. Supplemental Figure 5. Selected metabolism genes are not changed with GLUT5 overexpression. Supplemental Figure 6. Serum concentration of glucose overshadows fructose contributions to proliferation rate. Supplemental Figure 7. KHK overexpression does not rescue the ability to proliferate in fructose. Supplemental Figure 8. Trained PC3 have increased fructose flux into the TCA cycle. Supplemental Figure 9. Trained PC3 have increased fructose flux into the TCA cycle. Supplemental Table 1: Clinical and genomic data of profiled cell lines in order of fructolytic index. Related to Figure 1. Supplemental Table 2. qPCR data for each cell line using primers from Supplemental File 1. (n = 2 per gene per sample, 2^dCt values shown). Related to Figure 1. Supplemental Table 3. qPCR primers for selected metabolic genes, CRISPR-cas9 primers, and qPCR primers for gDNA. Related to Figures 1, 4 and Supplemental Figures 4, 8. Supplemental Table 4. qPCR primers for RNA-seq hits, related to Figure 3 and Supplemental Figure 4. [file 40170_2021_246_MOESM1_ESM.zip › SUPPLEMENTAL 9.tif]
